# Psychological Factors as Risk Contributors for Poor Hip Function after Periacetabular Osteotomy

**DOI:** 10.3390/jcm12124008

**Published:** 2023-06-12

**Authors:** Maximilian Fischer, Lars Nonnenmacher, Alexander Möller, André Hofer, Johannes Reichert, Georg Matziolis, Alexander Zimmerer, Georgi Wassilew

**Affiliations:** 1 Center for Orthopaedics, Trauma Surgery and Rehabilitation Medicine, University Medicine Greifswald, 17475 Greifswald, Germany; 2 Orthopedic Department, Jena University Hospital, Campus Eisenberg, 07607 Eisenberg, Germany; g.matziolis@waldkliniken-eisenberg.de; 3 Diakonieklinikum Stuttgart, Department of Orthopaedic and Trauma Surgery, Orthopädische Klinik Paulinenhilfe, 70176 Stuttgart, Germany

**Keywords:** hip dysplasia, acetabular retroversion, periacetabular osteotomy, hip preservation, psychometrics, psychological factors, depression, somatization, PROMs

## Abstract

Psychologic comorbidities have been identified as risk factors for poor outcomes in orthopedic procedures, but their influence on the outcome of hip-preserving periacetabular osteotomy (PAO) remains uncertain. This retrospective cohort study aimed to assess the impact of patients’ psychological health on the outcome of PAO in patients with hip dysplasia (HD) and acetabular retroversion (AR). The study included 110 patients undergoing PAO for HD or AR between 2019 and 2021. Standardized questionnaires were administered to assess psychological factors, postoperative hip function, and activity level (mean follow-up: 25 months). Linear regression analyses were used to examine the associations between psychological factors and postoperative hip function and activity level. Both HD and AR patients showed improved postoperative hip function and activity levels. Linear regression analyses revealed that depression significantly impaired postoperative outcomes in both groups, whereas somatization negatively influenced the outcome in AR patients. General health perceptions significantly contributed to an improved postoperative outcome. These findings highlight the importance of concomitantly addressing psychologically relevant factors in order to improve patient outcomes after PAO procedures. Future prospective studies should continue to investigate the impact of various psychological factors and explore possibilities of incorporating psychological support into routine postoperative care for these patient cohorts.

## 1. Introduction 

The Bernese periacetabular osteotomy (PAO), developed by Reinhold Ganz [1], has become a well-established surgical technique for the treatment of adult hip dysplasia (HD) and acetabular retroversion (AR) [2,3].

HD and AR are two main indications for this surgical procedure. The decreased coverage of the femoral head in HD can lead to hip joint instability and potentially to subluxation or dislocation. Acetabular retroversion, on the other hand, is a condition consisting of a malorientation of the acetabulum in the sagittal plane. This altered orientation can lead to an anterior impingement between the femoral head and the acetabulum. The prevalence of both hip pathologies ranges up to 20% in young adults [4,5]. 

The mainly young and active patients with high functional demand who have undergone PAO report intractable chronic hip pain, especially in the groin and lateral hip region. The pain can significantly impact their ability to engage in physical exercise, leading to a decline in their overall fitness and functional capacity [6]. 

While PAO has been shown to effectively improve hip function and to reduce pain, some patients still experience poor outcomes after the procedure [7,8]. Several factors have been identified as potential risk factors for poor outcomes after PAO, including advanced age, advanced osteoarthritis, obesity and intra-articular lesions [9,10]. Psychological factors, such as anxiety, depression and general health perception, have been identified as potential risk contributors promoting suboptimal outcomes after orthopedic procedures, particularly in knee and hip arthroplasty [11,12]. Other hip pathologies, such as hip dysplasia, are closely associated with various psychological comorbidities that have been reported. Thus, understanding the impact of a patient´s psychosocial status on the postoperative outcome could be the key for further improvements in patients’ care [13]. 

Several studies have reported that patients with preoperative psychological distress are more likely to experience poor outcomes after joint replacement, such as reduced function and higher rates of revision surgery [14,15,16,17,18]. However, the role of psychological factors in predicting outcomes after PAO, particularly in relation to differences in outcomes between PAO for hip dysplasia and PAO for acetabular retroversion, has mainly be unaccounted for in the literature [19]. 

Therefore, the purpose of this study was to investigate the relationship between psychological factors and hip function after PAO, with a focus on comparing outcomes between patients with HD and those with AR. We utilized the Brief Symptom Inventory (BSI) and the 36-item Short Form Survey (SF-36) as measures of psychological factors. Our patient-reported outcome measures (PROMs) included the Modified Harris Hip Score (mHHS), the International Hip Outcome Tool (iHOT–12), the University of California Los Angeles Activity Score (UCLA) and the Hip Disability and Osteoarthritis Outcome Score (HOOS).

Understanding the impact of psychological factors on outcomes after PAO in patients with different underlying conditions could help identify patients who may benefit from additional support to achieve optimal outcomes after surgery.

## 2. Patients and Methods

### 2.1. Study Design

We conducted a retrospective follow-up study of 248 patients who underwent PAO at our institution between January 2020 and December 2021. 

Institutional review board approval was obtained prior to the initiation of this study. Inclusion criteria for this specific analysis were as follows: PAO procedure ± femoral osteochondroplasty for HD or anteverting PAO ± femoral osteochondroplasty for AR. Exclusion criteria were lack of written informed consent, additional concomitant or subsequent procedures (e.g., femoral osteotomy, hip arthroscopy, surgical hip dislocation or contralateral PAO), revision PAO, and incomplete follow-up questionnaires. 

Of the 248 patients who underwent PAO in our clinic during the aforementioned period, 10 were excluded because of missing written informed consent, 5 as they underwent PAO due to diagnoses other than HD or AR, 89 because of subsequent or additional procedures (i.e., 60 contralateral PAOs, 18 hip arthroscopies, 4 surgical hip dislocations, 7 femoral rotational osteotomies), 6 who received revision PAO and 33 patients with incomplete follow-up data (only entirely complete datasets were included).

Of the remaining 110 patients (Table 1), 89 (81%) were diagnosed with hip dysplasia and 21 (19%) with acetabular retroversion. The general indication criteria for PAO in HD include a lateral center-edge angle (LCE Angle) of less than 25°, as measured by the modified Ogata CE angle [20], in combination with symptomatic, refractory hip pain lasting more than 6 months. Anteverting PAO was indicated in cases of radiographic AR (e.g., cross-over sign, posterior wall sign, and ischial spine sign) with a retroversion index >30% [21] and positive impingement signs on clinical examination combined with symptomatic, refractory hip pain persisting for more than 6 months. A general contraindication for both indications is radiologic evidence of advanced osteoarthritis (Toennis ≥ 2). The AR group solely included patients with pure acetabular retroversion (LCE Angle > 25°), whereas patients with hip dysplasia and frequent concomitant retroversion were included in the HD group. The mean preoperative LCE angle in the HD group was 17.8 ± 4.8° (mean ± SD), while in the AR group the mean LCE angle was 33.4 ± 5° (mean ± SD), with a mean retroversion index of 39.5 ± 8.7% (mean ± SD).

A modified, minimally invasive Bernese periacetabular osteotomy (PAO) technique was performed in all patients. This involved the use of a bikini incision and either a rectus-sparing (RS) approach with bony detachment of the sartorius from its origin or a rectus- and sartorius-sparing (RASS) approach, as previously described by our group [22]. All procedures were performed by the senior author (G.I.W). 

The mean age at surgery was 30.9 ± 8.18 years (mean ± SD), 26 patients (23.6%) were male, and 84 (76.4%) were female. The mean postoperative follow-up was 21 ± 7 months (mean ± SD). Psychological comorbidities were assessed at the preoperative consultation. Overall, 7.8% (7/89) of the patients in the HD group and 14.2% (3/21) of the AR group reported diagnosed psychological comorbidities. All of these patients were under pharmacological treatment for psychological comorbidities prior to the PAO. Before the surgery, chronic pain syndromes other than that of the hip joint were reported in 10.1% (9/89) of patients with HD and in 19% (4/21) of patients with AR.

### 2.2. Data Collection

Data was collected from electronic medical records, including patients’ demographics, preoperative comorbidities and operative details. The functional outcome after surgery was assessed using a standardized postoperative questionnaire which included the Modified Harris Hip Score (mHHS), the International Hip Outcome Tool (iHOT–12), the University of California Los Angeles Activity Scale (UCLA) and the Hip Disability and Osteoarthritis Outcome Score (HOOS). Psychological parameters were assessed by the Brief Symptom Inventory 18 (BSI-18) and the 36-Item Short Form Survey (SF-36).

The BSI-18 consists of 18 items on a 5-point (0–4) Likert scale, and is designed to assess current psychological distress in adult patients over an age of 20 [23]. In addition to a total score, referred to as the global severity index (GSI; max = 72), separate scores can be calculated on three subscales: “somatization”, “depression”, and “anxiety”, with six questions contributing to each subscale (max = 24). Higher scores reflect greater distress [24].

The SF-36 is a comprehensive measure that includes 36 items which assess eight domains of health-related quality of life: physical functioning, role physical, bodily pain, general health, vitality, social functioning, role emotional and mental health [25]. There are two distinct concepts measured by the SF-36: a physical dimension, represented by the physical component summary (PCS), and a mental dimension, represented by the mental component summary (MCS) [26].

### 2.3. Statistical Analysis

Descriptive statistics were used to summarize the patients’ characteristics and outcomes. Data were reported as means and standard deviations. Statistical analysis and dataset presentation were performed using GraphPad Prism 9.4.1. A two-tailed paired *t*-test was used to compare preoperative and postoperative functional outcomes. Pearson correlations were used to identify the impact of patients’ psychological parameters on postoperative hip function. Each psychological parameter (i.e., “general health perception”, “depression”, “psychological well-being”, “somatization” and “anxiety”) was individually correlated with the postoperative mHHS, iHot–12 and UCLA scores. For HOOS score analyses, each psychological parameter was individually correlated with the HOOS subscales “symptom”, “pain”, “activity in daily life”, “sport” and “quality of life”. A *p*-value less than 0.05 was considered statistically significant. The Pearson correlation coefficient was interpreted as very weak (±0.1–0.19), weak (±0.2–0.39), moderate (±0.4–0.59), strong (±0.6–0.79), or very strong (±0.8–1).

### 2.4. Ethical Considerations

All patients gave written informed consent before inclusion. Ethics approval (BB 099/20) was obtained from the local independent ethics committee (IEC) of the University Medicine Greifswald according to the World Medical Association Declaration of Helsinki.

## 3. Results

The study aimed to investigate the impact of a patient´s psychological health on their functional outcome and activity level after PAO for HD or AR.

**Figure 1:**
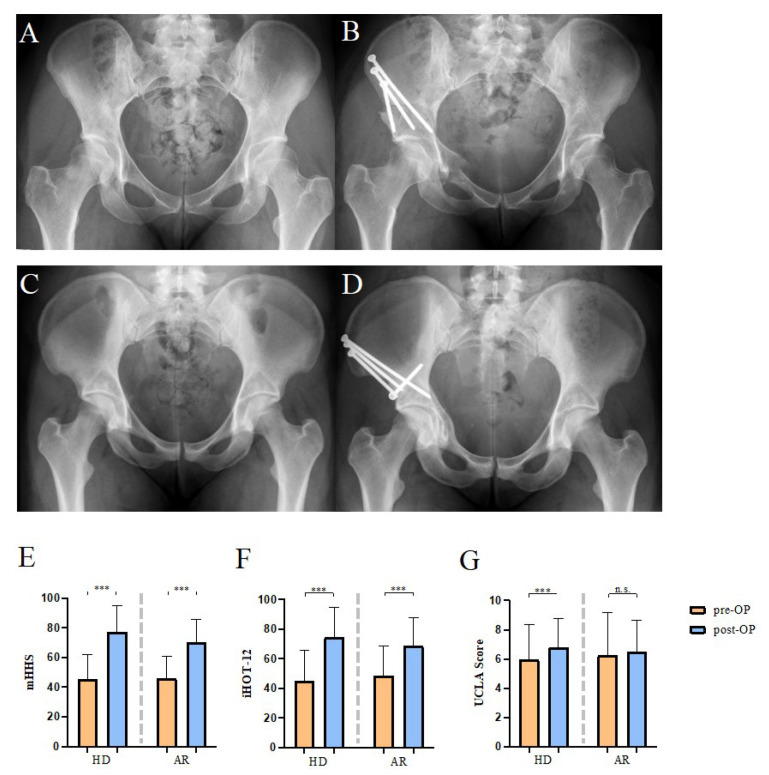
Hip functionality and activity analysis indicates improved postoperative scores after PAO for HD and AR. (**A**,**B**) Representative X-ray (pelvic AP view) before and after PAO due to HD. (**C**,**D**) Representative X-ray (pelvic AP view) before and after PAO to correct AR. (**E**) MHHS scores indicate an increase of hip functionality after PAO in HD and AR patients. (**F**) PAO improved the health-related quality of life in patients with HD and AR. (**G**) Higher UCLA-score in the HD but not in the AR group indicates improved physical activity in HD patients after PAO in comparison to pre-operative conditions. HD, Hip dysplasia; AR, Acetabular retroversion. (Mean ± SD, two-tailed paired *t*-test, levels of significance: *** *p* < 0.001, n.s. not significant).

First, we analyzed hip function and activity levels prior to and 12 months after surgery using the mHHS, iHOT–12, UCLA and HOOS subscores (Figure 1). The mHHS increased significantly after PAO for HD (MD 31.7, *p* < 0.001) and AR (MD 24.1, *p* < 0.001). Moreover, the iHOT–12 also showed a significant increase in the HD (MD 29.3, *p* < 0.001) and AR groups (MD 20.1, *p* < 0.001). As to the UCLA, only HD patients (MD 0.86, *p* < 0.001) had a significant higher score postoperatively compared to their preoperative condition. For both investigated indications, the postoperative HOOS score was significantly higher after PAO, with a higher increase in the HD group (MD 25.43, *p* < 0.001) as compared to the AR group (MD 15.8, *p* = 0.005).

### 3.1. Patients’ Psychological Health Influences Postoperative Hip Function and Activity in HD and AR

Having shown that PAO significantly improves hip function and the activity levels of patients with HD and AR, the aim was to analyze the impact of patients’ psychological parameters on postoperative hip functionality and activity scores.

“General health perception” showed a strong positive correlation with the postoperative mHHS in patients with HD (Pearson r = 0.66, *p* < 0.001), as well as a moderate positive correlation with the postoperative iHOT–12 (Pearson r = 0.58, *p* < 0.001) and UCLA (Pearson r = 0.47, *p* < 0.001) scores within this cohort.

In contrast, “depression” was closely associated with worse postoperative outcomes in HD patients, indicated by a strong negative correlation with the postoperative mHHS (Pearson r = −0.68, *p* < 0.001) and a moderate negative correlation with the postoperative iHOT–12 (Pearson r = −0.59, *p* < 0.001) and UCLA scores (Pearson r = −0.58, *p* < 0.001).

Analyzing the impact of the “psychological well-being”, there was a weak positive correlation (Pearson r < 0.40) with the postoperative hip and activity scores (Table 2).

Moreover, the study aimed to evaluate the influence of self-reported “somatization” and “anxiety” levels on the outcome after surgery in HD patients. The data showed, for both psychological parameters, poorer postoperative hip function and activity levels with increasing “somatization” and “anxiety” levels. For all analyzed postoperative hip scores, the data showed a weak negative correlation with the patients self-reported “anxiety” level (−0.20 < Pearson r > −0.39) (Table 2).

“Somatization” showed a weak negative correlation with postoperative mHHS, iHOT–12 and UCLA scores (Table 2).

Having identified “general health perception” and “depression” as main psychological factors affecting the clinical outcome after PAO for HD, the question was whether the same psychological aspects related to the PAO outcome in patients suffering from AR.

In line with HD outcome scores, “general health perception” showed a significant positive correlation with all analyzed postoperative hip scores in AR. There was a strong positive correlation for postoperative mHHS (Pearson r = 0.66, *p* < 0.01), iHOT–12 (Pearson r = 0.60, *p* < 0.01) and UCLA scores (Pearson r = 0.61, *p* < 0.01) in relation to the patient´s self-reported “general health perception” in the AR group.

“Depression” showed a significant impact on postoperative hip function and daily activity in patients with AR. In line with the results for HD patients, there was a moderate negative correlation between the “depression” score and scores for postoperative mHHS (Pearson r = −0.43, *p* < 0.05), iHOT–12 (Pearson r = −0.40, ns) and UCLA (Pearson r = −0.49, *p* < 0.05).

In contrast to HD patients, “somatization” showed a strong impact on AR patients’ outcome after PAO. Higher “somatization” levels were related to poorer postoperative hip function and activity levels in patients with AR, as indicated by the strong negative correlation with scores for mHHS (Pearson r = −0.70, *p* < 0.001) and iHOT–12 (Pearson r = −0.63, *p* < 0.01).

“Anxiety” correlated only weakly and negatively with the postoperative hip and activity scores (Table 2). As already indicated for HD patients’, “psychological well-being” correlated positively with the outcome after PAO. The data showed a moderate correlation with the postoperative UCLA score (Pearson r = 0.40, ns) and moderate correlations with the mHHS and iHOT–12 scores, indicating improved postoperative hip function and activity level in relation to the greater “psychological well-being” of patients with AR (Table 2).

### 3.2. HOOS Subscale Analysis

Next, the postoperative HOOS subscales “symptom”, “pain”, “activity in daily life”, “sport” and “quality of life” were individually correlated with the previously mentioned psychological parameters. The results are presented in Table 3.

Significant negative correlations were found in the HD group between the HOOS “symptom” subscale and depressive symptoms (r = −0.55, *p* < 0.001), as well as between the HOOS “pain” subscale and depressive symptoms (r = −0.65, *p* < 0.001). In addition, significant negative correlations were found between the HOOS “symptom” and “pain” subscales and “anxiety” (r = −0.32 and r = −0.3, respectively, both *p* < 0.05). The HOOS “symptom” subscale was also positively correlated with “general health perception” (r = 0.41, *p* < 0.001), as was the HOOS “pain” subscale (r = 0.41, *p* < 0.001) and the HOOS “ADL” subscale (r = 0.37, *p* < 0.001). The HOOS “QoL” subscale was positively correlated with “general health perception” (r = 0.29, *p* < 0.01). Significant, negative correlations between “somatization” and the HOOS subscales were only found for HOOS “symptom”, “pain” and “QoL” (*p* < 0.05), with no significant correlation between “somatization” and HOOS “ADL” and “sport.” Similarly, no significant correlations were found between “anxiety” and the HOOS “sport” and “QoL” subscales.

The HOOS subscale analysis in the HD group consistently showed significant and moderate to strong correlations with the depression scores of the psychological parameters.

In the AR group, strong positive correlations were found between “general health perception” and the HOOS “pain” (r = 0.54, *p* < 0.01), “ADL” (r = 0.55, *p* < 0.01), and “QoL” (r = 0.71, *p* < 0.001) subscales. Strong negative correlations were found between “somatization” and all HOOS subscales. No other significant correlations were found between HOOS subscales and the psychological variables measured in the AR group.

### 3.3. Conclusions

Taken together, the data underline that the postoperative outcome after PAO is significantly affected by patient´s “psychological health” in both cohorts. Comparing HD and AR, there are differences in psychological risk factors affecting postoperative hip function and activity level. “Depression” was negatively correlated with postoperative hip function and activity levels in both groups, while “somatization” demonstrated a significantly negative correlation with postoperative outcome only in patients with AR. “General health perception” was the only psychological parameter analyzed that showed a significant positive correlation with the postoperative outcome in both HD and AR patients.

## 4. Discussion

The purpose of this retrospective study was to evaluate the impact of psychological health on postoperative outcomes following hip-preserving periacetabular osteotomy (PAO) for hip dysplasia (HD) and acetabular retroversion (AR) and to do a comparative analysis between both main indications.

Taken together, the study results indicate that both HD and AR patients had improved hip function and activity levels postoperatively. However, the results also suggest that psychological health is an important factor determining PAO outcomes, as indicated by several patient-reported outcome measures (PROMs), including the Modified Harris Hip Score (mHHS), the International Hip Outcome Tool (iHOT–12), and the University of California Los Angeles Activity Score (UCLA), as well as subscales of the Hip Disability and Osteoarthritis Outcome Score (HOOS). Specifically, “depression” was found to be strongly associated with worse postoperative outcomes in both groups, while “somatization” negatively impacted the outcome in AR patients only. On the other hand, general health perception was closely associated with improved postoperative hip function and activity levels.

PAO has been reported to favor significant improvements in pain relief, hip function and patient satisfaction. Several studies have shown that PAO results in increased joint stability, improved hip function and long-term preservation of the hip joint in both HD and AR patients [27,28]. However, the success of PAO depends on various factors, including well-selected patients or experienced surgeons with a refined surgical technique [29].

Psychologic comorbidities have been reported as risk factors for a poor outcome after elective orthopedic surgeries [11]. In this study, 7.8% of patients in the HD group and 14.2% of patients in the AR group reported medically diagnosed and treated psychological comorbidities before the surgical treatment. This is in line with the reported prevalence of psychological disorders in young adults in the general population and does not indicate for a higher psychological risk profile within the study population preoperatively [30].

Consistent with the findings of the current study, studies in different types of orthopedic surgery, such as total joint replacement or spine surgery, have emphasized that depression was significantly associated with increased postoperative pain levels and reduced functional outcomes [31,32,33]. Thus, addressing psychological factors such as depression may be critical to improving outcomes in patients undergoing orthopedic surgery.

Indeed, there is limited evidence regarding the specific role of psychological factors in relation to postoperative outcomes of HD and AR. Previous studies have primarily focused on general orthopedic procedures or other orthopedic conditions. Gudmundsson et al. suggest that improved hip function after therapy for HD may have a positive impact on psychological comorbidities; this is caused by the association between joint function and psychological factors, and the authors underline the importance of considering mental health as a treatment target in multidisciplinary approaches for orthopedic surgery [19]. Indeed, Brandon et al. showed, in a study of 48 patients undergoing PAO for HD, an improvement with respect to depression, anxiety and pain catastrophizing postoperatively [34]. In the field of other joint-preserving procedures, a recent case-control study of 77 patients undergoing hip arthroscopy identified depression as a risk factor for poorer postoperative outcomes [35]. The authors concluded that patient well-being is an important contributor to attaining satisfying postoperative outcomes. This is in accordance with the results of this study, in which an association between postoperative hip function and activity levels and psychologic parameters was found. 

In addition, in our study, somatization was associated with a worse outcome in AR patients. Somatization is defined as a psychological condition in which physical symptoms are experienced as a result of emotional or psychological distress. The influence of somatization on postoperative outcomes after orthopedic surgery has been demonstrated previously [36,37]. However, it has now additionally been identified as a contributing factor for poorer outcomes following joint-preserving hip surgery.

Furthermore, the present study also demonstrates that general health perception is linked to improved postoperative outcomes in both groups of patients undergoing PAO. This suggests that a patient's overall perception of health is an important factor in determining the postoperative outcome. It is possible that patients who have a positive outlook on their health are more likely to have a better recovery after surgery [38].

Orthopedic surgery is able to improve the general health-perception and quality of life postoperatively [39]. Nevertheless, it remained unknown whether a higher SF-36-measured general health-perception was associated with improved postoperative hip function and activity level after PAO. With our study results, we provide actual evidence for this interrelationship.

Overall, the findings of our study highlight the importance of considering psychological factors in the management of patients undergoing PAO. The data suggest that different psychological risk factors impact the outcome after PAO in patients diagnosed with HD and AR. Consequently, the current findings may have wide and important implications for the future clinical management of patients undergoing PAO, their preoperative evaluation, and postoperative care after identification of these specific risk factors.

However, several limitations of this study should be acknowledged. Firstly, the retrospective design of the study and the use of PROMs to evaluate outcomes after PAO have to be mentioned.

Due to the retrospective study design, important factors such as the preoperative emotional state, depression and health characteristics were not assessed. These factors can potentially influence the outcome and may provide a more comprehensive understanding of the relationship between psychological factors and postoperative results, and have to be considered when evaluating the study’s results.

Additionally, the sample size was relatively small, with a notable difference in the number of patients between the two groups (89/21). While the findings from this pilot study provide valuable preliminary insights, the limited sample size restricts the generalizability of the results.

Future studies with a larger sample size will allow for more robust subgroup analyses and multivariate analyses to investigate the independent effects of psychological factors on the postoperative outcomes. Another important limitation of our study is that the analysis primarily focused on examining correlations between different variables. While significant associations between certain factors were observed, it is essential to acknowledge that correlation does not imply causation. Although our findings provide valuable insights for the interrelationship of these variables, they do not establish a cause-and-effect relationship. Future research utilizing experimental designs or longitudinal studies is warranted to further explore the causal mechanisms underlying these associations.

Therefore, further prospective studies with systematic assessment of preoperative psychological factors are necessary to better understand these relationships and develop more targeted preoperative and/or postoperative measures. It is essential to address these limitations in future research to enhance the validity and generalizability of our findings.

## Figures and Tables

**Table 1:** Patients’ characteristics.

	Hip Dysplasia	Acetabular Retroversion
Number of patients	89	21
Age (mean ± SD)	31.7 ± 7.98	27.3 ± 8.0
Sex (% female)	77.5	71.4
Psychological comorbidity pre OP (%) Under pharmacological treatment (%)	7 (7.8) 7 (100)	3 (14.2) 3 (100)
Chronic pain syndrome pre OP (%)	9 (10.1)	4 (19)
Pre LCE angle (° ± SD) Max–Min (°)	17.8 ± 4.78 26–5	33.4 ± 4.97 42–25
Post LCE angle (° ± SD) Max–Min (°)	30.1 ± 3.75 39–17	33.5 ± 4.29 39–26
Pre retroversion index (%° ± SD)	n.a.	39.5° ± 8.72
Post retroversion index (%° ± SD)	n.a.	16.1° ± 9.83

LCE, lateral center edge angle; SD, standard deviation, n.a. not available.

**Table 2:** “Depression” showed an inverse correlation with the postoperative outcome after PAO in HD and AR patients, while “somatization” negatively impacted the outcome only in AR patients. “General health perception” was associated with an improved postoperative outcome in both groups.

	HD (Pearson r)	AR (Pearson r)
**SF36/BSI**	**mHHS**	**mHHS**
General health perception	0.66 *(p < 0.001)*	0.66 *(p < 0.01)*
Depression	−0.68 *(p < 0.001)*	−0.43 *(p < 0.05)*
Psychological well-being	0.34 *(p < 0.001)*	0.38 *(n.s.)*
Somatization	−0.18 *(n.s.)*	−0.7 *(p < 0.001)*
Anxiety	−0.28 *(p < 0.01)*	−0.27 *(n.s.)*
**SF36/BSI**	**iHOT–12**	**iHOT–12**
General health perception	0.58 *(p < 0.001)*	0.6 *(p < 0.01)*
Depression	−0.59 *(p < 0.001)*	−0.4 *(n.s.)*
Psychological well-being	0.30 *(p < 0.01)*	0.39 *(n.s.)*
Somatization	−0.21 *(p < 0.05)*	−0.63 *(p < 0.01)*
Anxiety	−0.27 *(p < 0.05)*	−0.26 *(n.s.)*
**SF36/BSI**	**UCLA**	**UCLA**
General health perception	0.47 *(p < 0.001)*	0.61 *(p < 0.01)*
Depression	−0.58 *(p < 0.001)*	−0.49 *(p < 0.05)*
Psychological well-being	0.36 *(p < 0.001)*	0.4 *(n.s.)*
Somatization	−0.12 *(n.s.)*	−0.16 *(n.s.)*
Anxiety	−0.3 *(p < 0.05)*	−0.1 *(n.s.)*

HD, Hip dysplasia; AR, Acetabular retroversion; mHHS, Modified Harris Hip Score; iHOT–12, International Hip Outcome Tool; UCLA, University of California Los Angeles Activity Score; HOOS, Hip Disability and Osteoarthritis Outcome Score; BSI, Brief Symptom Inventory; SF-36, Short Form Survey, *n.s.* not significant. (Mean ± SD; Pearson correlation r).

**Table 3:** HOOS subscale analyses showed “depression”, “general health” and “somatization” as main psychological factors associated with the postoperative outcome after PAO in HD and AR.

	HD (Pearson r)	AR (Pearson r)
**SF36/BSI**	**HOOS symptom**	**HOOS symptom**
General health perception	0.41 (*p < 0.001*)	0.35 (*n.s.*)
Depression	−0.55 (*p < 0.001*)	−0.11 (*n.s.*)
Psychological well-being	0.32 (*p < 0.05*)	0.12 (*n.s.*)
Somatization	−0.22 (*p < 0.05*)	−0.57 (*p < 0.01*)
Anxiety	−0.32 (*p < 0.05*)	−0.22 (*n.s.*)
**SF36/BSI**	**HOOS pain**	**HOOS pain**
General health perception	0.41 (*p < 0.001*)	0.54 (*p < 0.01*)
Depression	−0.65 (*p < 0.001*)	−0.19 (*n.s.*)
Psychological well-being	0.33 (*p < 0.05*)	0.22 (*n.s.*)
Somatization	−0.29 (*p < 0.05*)	−0.60 (*p < 0.001*)
Anxiety	−0.30 (*p < 0.05*)	−0.22 (*n.s.*)
**SF36/BSI**	**HOOS activity in daily life**	**HOOS activity in daily life**
General health perception	0.37 (*p < 0.001*)	0.55 (*p < 0.01*)
Depression	−0.62 (*p < 0.001*)	−0.31 (*n.s.*)
Psychological well-being	0.27 (*p < 0.05*)	0.35 (*n.s.*)
Somatization	−0.20 (*n.s.*)	−0.68 (*p < 0.001*)
Anxiety	−0.28 (*p < 0.05*)	−0.29 (*n.s.*)
**SF36/BSI**	**HOOS sport**	**HOOS sport**
General health perception	0.35 (*p < 0.001*)	0.42 (*n.s.*)
Depression	−0.56 (*p < 0.001*)	−0.21 (*n.s.*)
Psychological well-being	0.21 (*p < 0.05*)	0.20 (*n.s.*)
Somatization	−0.17 (*n.s.*)	−0.61 (*p < 0.01*)
Anxiety	−0.17 (*n.s.*)	−0.09 (*n.s.*)
**SF36/BSI**	**HOOS Quality of life**	**HOOS quality of life**
General health perception	0.29 (*p < 0.01*)	0.71 (*p < 0.001*)
Depression	−0.51 (*p < 0.001*)	−0.19 (*n.s.*)
Psychological well-being	0.24 (*p < 0.05*)	0.20 (*n.s.*)
Somatization	−0.22 (*p < 0.05*)	−0.52 (*p < 0.01*)
Anxiety	−0.15 (*n.s.*)	−0.19 (*n.s.*)

HD, Hip dysplasia; AR, Acetabular retroversion; HOOS, Hip Disability and Osteoarthritis Outcome Score; BSI, Brief Symptom Inventory; SF-36, Short Form Survey, *n.s.* not significant. (Mean ± SD, Pearson correlation r).

## Data Availability

Not applicable.
